# Menthol: An underestimated anticancer agent

**DOI:** 10.3389/fphar.2023.1148790

**Published:** 2023-03-17

**Authors:** Yijia Zhao, Huafeng Pan, Wei Liu, E. Liu, Yaobin Pang, Hongjin Gao, Qingying He, Wenhao Liao, Yejing Yao, Jinhao Zeng, Jing Guo

**Affiliations:** ^1^ Dermatological Department, Hospital of Chengdu University of Traditional Chinese Medicine, Chengdu, China; ^2^ Science and Technology Innovation Center, Guangzhou University of Chinese Medicine, Guangzhou, China; ^3^ The First Affiliated Hospital, Guangzhou University of Chinese Medicine, Guangzhou, China; ^4^ Department of Gastroenterology, Hospital of Chengdu University of Traditional Chinese Medicine, Chengdu, China; ^5^ TCM Regulating Metabolic Diseases Key Laboratory of Sichuan Province, Hospital of Chengdu University of Traditional Chinese Medicine, Chengdu, China

**Keywords:** anticancer, menthol, natural product, molecular mechanism, future direction

## Abstract

Menthol, a widely used natural, active compound, has recently been shown to have anticancer activity. Moreover, it has been found to have a promising future in the treatment of various solid tumors. Therefore, using literature from PubMed, EMBASE, Web of Science, Ovid, ScienceDirect, and China National Knowledge Infrastructure databases, the present study reviewed the anticancer activity of menthol and the underlying mechanism. Menthol has a good safety profile and exerts its anticancer activity *via* multiple pathways and targets. As a result, it has gained popularity for significantly inhibiting different types of cancer cells by various mechanisms such as induction of apoptosis, cell cycle arrest, disruption of tubulin polymerization, and inhibition of tumor angiogenesis. Owing to the excellent anticancer activity menthol has demonstrated, further research is warranted for developing it as a novel anticancer agent. However, there are limitations and gaps in the current research on menthol, and its antitumor mechanism has not been completely elucidated. It is expected that more basic experimental and clinical studies focusing on menthol and its derivatives will eventually help in its clinical application as a novel anticancer agent.

## 1 Introduction

Cancer is one of the major health conditions that negatively impact human life and health, and its incidence is increasing annually. According to the Cancer Surveillance Branch at the International Agency for Research on Cancer, an estimated 19.3 million people worldwide were newly diagnosed with cancer, with 10 million cancer-related deaths in 2020. In addition, the risk of developing cancer in a lifetime (before 75 years) is 20%, with the risk of dying from cancer being 10% (Ferlay et al., 2021). This means that approximately 20,000 out of 100,000 people will get cancer in their lifetime, and 10,000 of them will die. Currently, surgery, radiotherapy, immunotherapy, and targeted therapy are the primary modalities for treating cancer. However, these available treatments cannot completely treat or eliminate cancer and are associated with considerable toxic side effects, sequelae. More importantly, the economic burden of cancer is profound on both patients and their families. Altice *et al.* reported that in the United States, the direct cost of oncology treatment ranges from $316 to $741 per month, with 12%–64% of survivors facing debt due to treatment expenses, 47%–49% experiencing financial hardship, and 4%–45% discontinuing treatment owing to high cost (Altice et al., 2016). In addition, the financial losses incurred by patients’ families owing to patient care and treatment are becoming a social issue of concern (Alzehr et al., 2022). Chemotherapy, immunotherapy, gene therapy, and radiation therapy are the mainstay of cancer treatment (Malik et al., 2021). However, these are associated with low cure rates, prohibitive costs, and numerous residual effects. Owing to these limitations of the existing treatment options, researchers have been constantly searching for new therapeutic strategies. Natural phytochemicals have been proven to play a key role in the prevention and treatment of diseases, especially various types of cancer. Paclitaxel is one such natural phytochemical that has been successfully used as an anticancer drug. Thus, owing to their rich biological origin and safety, an increasing number of researchers have been attempting to synthesize novel anticancer drugs from natural products. From 1981 to 2019, 1,881 natural phytochemicals, including 247 anticancer drugs, were approved for therapeutic purposes, and the number continues to increase (Newman and Cragg, 2020). Among these natural products, monoterpenes offer distinct advantages in cancer treatment (Zielińska-Błajet et al., 2021).

**FIGURE 1:**
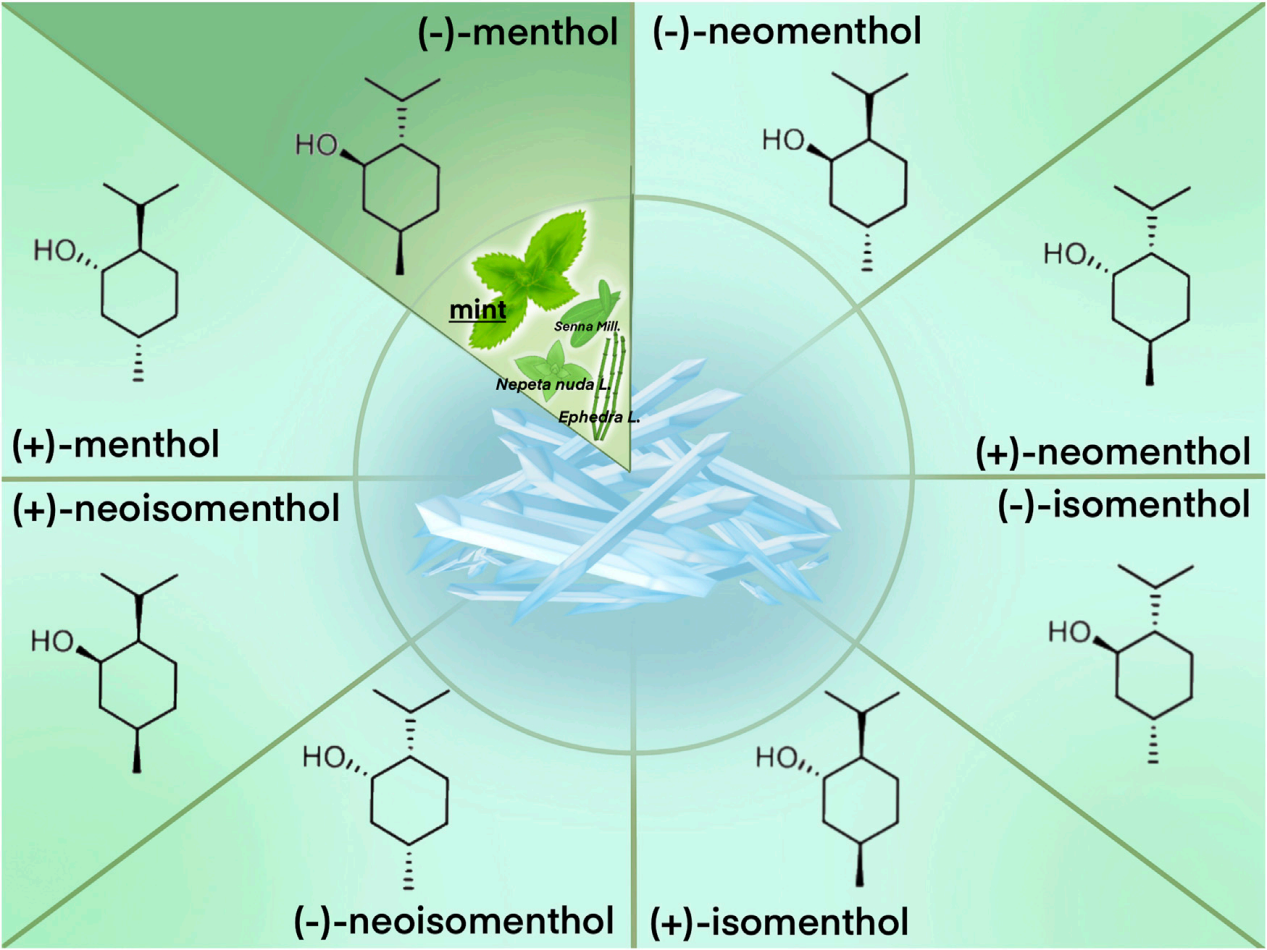
Plant sources of menthol and isomer’s structure.

Menthol (*5-methyl-2-propan-2-ylcyclohexan-1-ol*), also known as mint camphor, is a cyclic monoterpene alcohol derived mainly from mint and aromatic plants, such as *Nepeta nuda* L. and those belonging to the genera *Senna* Mill. And *Ephedra* L. It is commonly used in food and healthcare products (Cohen et al., 2020). Menthol possesses three stereo genic centers and thus has four pairs of optical isomers (+)- and (−)-menthol, (+)- and (−)-neomenthol, (+)- and (−)-neoisomenthol, and (+)- and (−)-isomenthol (Gpp et al., 2013; Zielińska-Błajet et al., 2021). The most common, naturally found isomer of menthol found is (−)-menthol (L-menthol), with 1R, 3R, 4S configuration. At room temperature (25°C), menthol is a white or colorless, flaky, solid, crystalline substance, with a density of 0.890 kg/dm^3^ and a melting point of 41°C−44°C depending on its purity. Menthol is not entirely soluble in water (435.5 mg/L at 25°C) but is freely soluble in alcohol, diethyl ether, and chloroform (Gpp et al., 2013). Notice that when menthol is mentioned alone, it usually refers to all isomers in general, or (−)-menthol (also known as L-menthol) if mentioned for experiments, and so in this paper (Figure 1).

**FIGURE 2:**
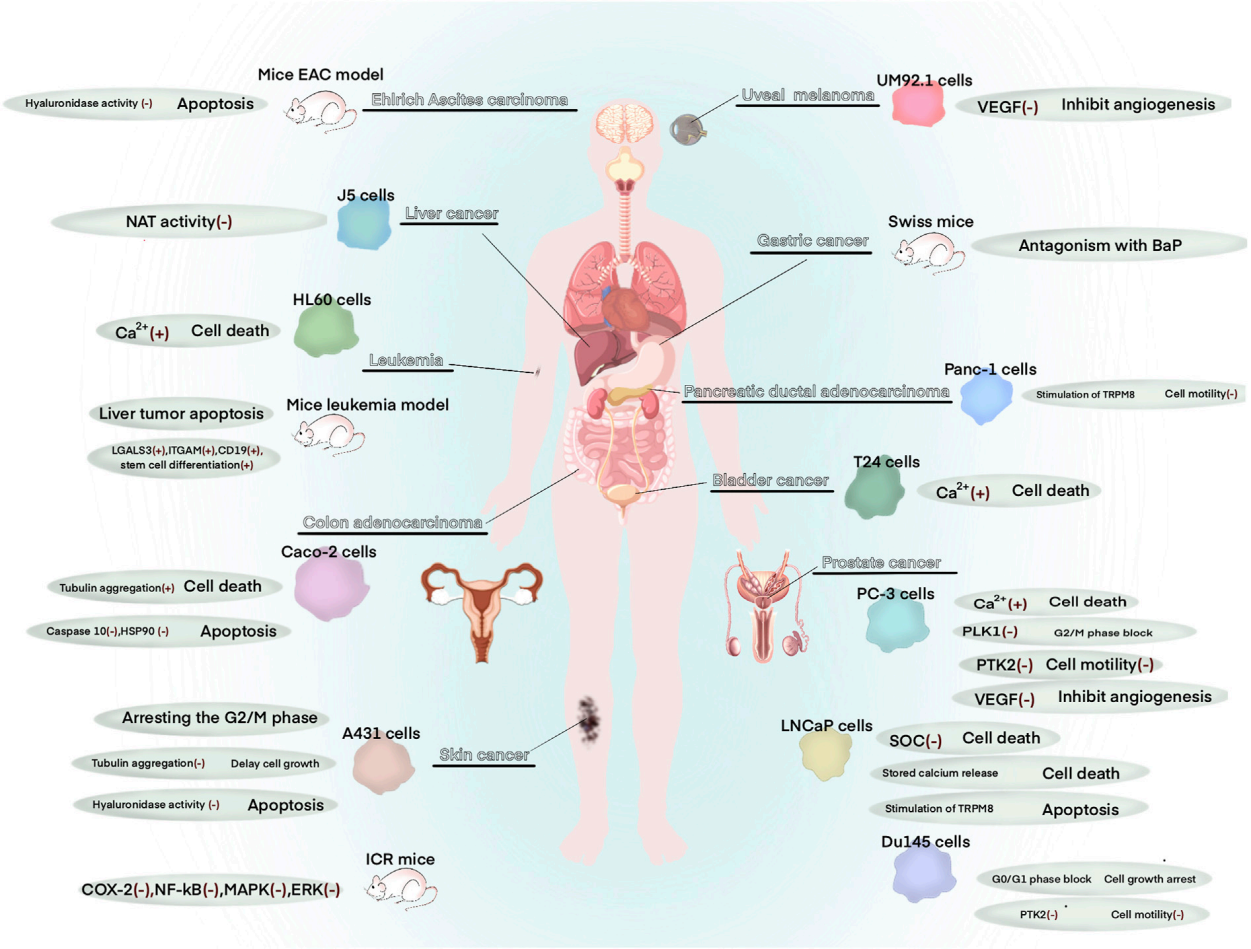
Role of menthol in a variety of human cancers.

Owing to its numerous biological properties, menthol is extensively used in multiple diseases, including inflammatory diseases (Du et al., 2020; Zhang et al., 2020), cancer (de Mesquita et al., 2019; Zielińska-Błajet et al., 2021), pain disorders (Hilfiger et al., 2021), respiratory disorders (Kanezaki et al., 2021), cardiovascular diseases (Silva, 2020), and skin diseases (Chrisman, 1978; Patel and Yosipovitch, 2010). Particularly, *in vitro* experiments have revealed the anti-proliferative potential of menthol against various tumor cell lines (Fatima et al., 2021). Mechanistically, menthol induces apoptosis in cancer cells, indicating its role as an anticancer agent (Beck et al., 2007) against a variety of cancers, such as prostate cancer (Kim et al., 2009), colon cancer (Faridi et al., 2016), skin cancer (Fatima et al., 2021), uveal melanoma (Walcher et al., 2018), pancreatic ductal adenocarcinoma (Cucu et al., 2014), gastric cancer (Santo et al., 2021), liver cancer (Lin et al., 2001), leukemia (Lu et al., 2007a), and bladder cancer (Li et al., 2009) (Figure 2). Irrespective of these data, the anticancer properties of menthol have not been sufficiently implemented in clinical practice. Moreover, no study has systematically reviewed the available anticancer data on menthol. Accordingly, the present article reviews advances in research on the anticancer properties of menthol as well as the underlying mechanisms *in vivo* and *in vitro*. In addition, the current anticancer applications and limitations of menthol in clinical practice are mentioned.

## 2 Menthol and its selective isomers, and the related derivatives

Natural menthol is mainly obtained from cornmint oil that is produced by steam distillation, and the menthol content of cornmint oil is 55%–85% (Gpp et al., 2013). Alternative methods to the expensive and inefficient distillation of cornmint oil have been attempted over the years, and synthesis was the first method practiced by scientists. The synthesis steps of menthol are relatively complex, condensation of isopentenyl diphosphate and dimethyl allyl pyrophosphate yields the universal monoterpene precursor geranyl diphosphate, which is then converted to (−)-limonene by cyclization. (−)-Limonene is converted to (−)-trans-isopentenol by hydroxylation with NADPH- and oxygen-dependent. (−)-Isothiopentenone is formed by allylic oxidation followed by NADPH-dependent reduction to (+)-cis-isothiopentenone. The (+)-cis-isopentanone was isomerized to form (+)-isopentanone, a precursor of (+)-menthofuran, (−)-menthone and (+)-isopentanone. Reduction of these ketones gave (−)-menthol, (+)-neomenthol, (+)-isomenthol and (+)-neoisomenthol (Croteau et al., 2005).

Due to the disadvantages of menthol’s volatility and short duration of action, there are more researchers have started to focus on the synthesis of menthol derivatives, such as menthyl lactate (Api et al., 2018), menthyl acetate (Api et al., 2017), Menthone 1, 2-glycerol ketal(FrescolatMGA), etc., Each of these derivatives builds on the strengths of menthol by being less irritating, evaporating more slowly or retaining their fragrance longer (Api et al., 2017; Api et al., 2018). These early menthol derivatives were mostly used in the household products and cosmetics industries, but as the medical value of menthol has received more attention, scientists have come to expect more from the derivatives. Attempts have been made to combine menthol with other known effective groups or substances to obtain a new compound that incorporates the advantages. For instance, menthol sulfamate and menthol carbonyl sulfamate, synthetic derivatives of sulfamate and menthol, have been shown to have a significant positive effect on Alzheimer’s disease (Daryadel et al., 2018). Menthol carbonates synthesized by Clemente’s team were validated to have anti-parasitic activity and have the potential to be orally active drug candidates (Clemente et al., 2022). In general, similar to menthol itself, menthol derivatives have also been found to have anti-cancer effects (Singh et al., 2022).

## 3 Mechanism underlying the anticancer activity of menthol

**FIGURE 3:**
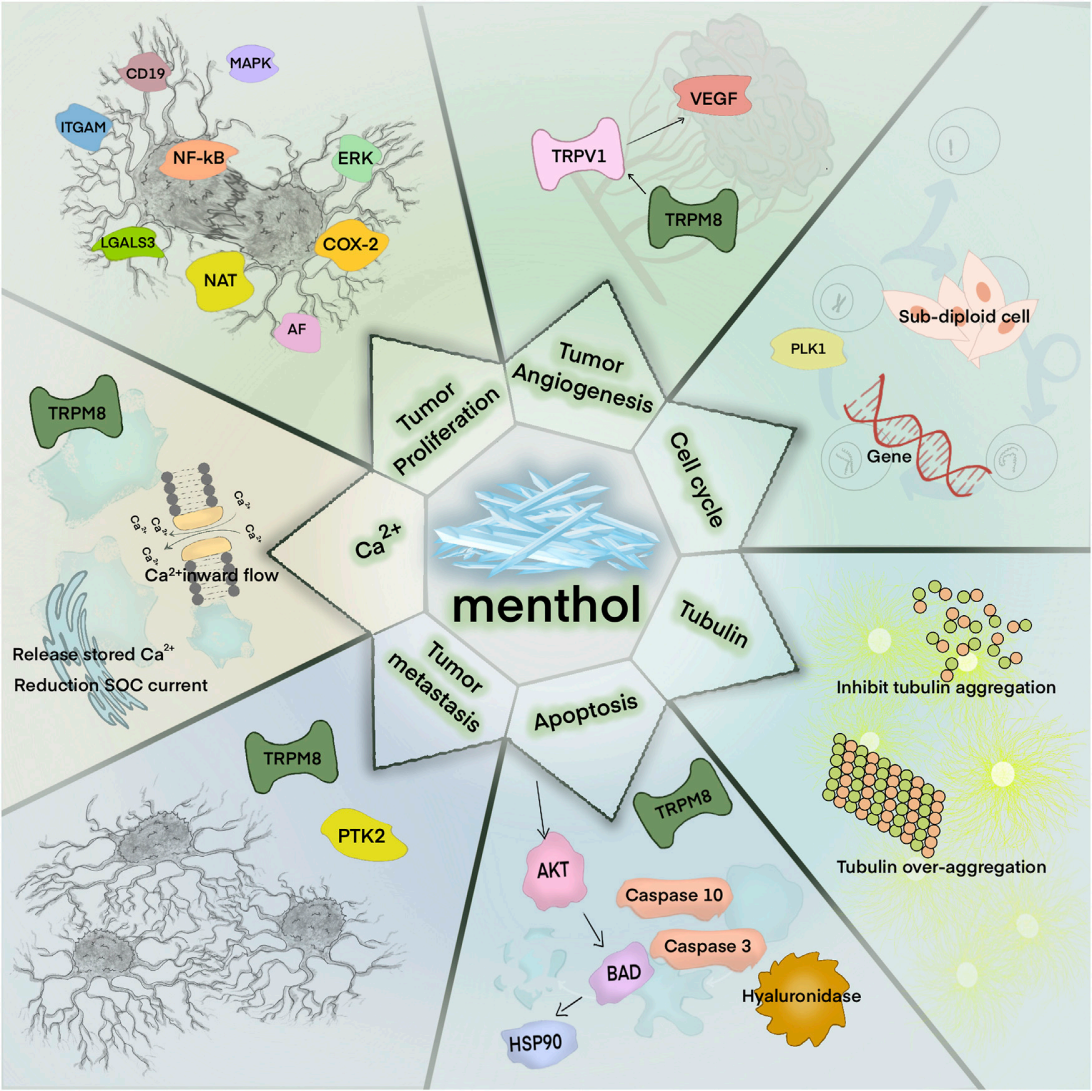
Anticancer strategy of menthol.

Over the years, both the anticancer targets and mechanisms underlying the anticancer activity of natural products have been extensively studied. Menthol exerts its anticancer activity by inducing cancer cell death *via* apoptosis, cell cycle arrest, inhibiting tubulin polymerization, and cell necrosis as well as by inhibiting tumor cell invasion, metastasis, angiogenesis, and proliferation (Figure 3; Table1).

**TABLE 1:** Mechanisms underlying the anticancer activity of menthol.

Cellular, biochemical, and molecular mechanisms		Diseases	Cell line	Isomer	References
Induction of cancer cell apoptosis	Via inhibition of hyaluronidase activity	Skin cancer	A431 cells	Neomenthol	Fatima *et al.* Fatima et al. (2021)
	Via inhibition of hyaluronidase activity	Ehrlich ascites carcinoma	Ehrlich ascites carcinoma mouse model	Neomenthol	Fatima et al. Fatima et al. (2021)
	Via modulation of heat shock protein 90 and activation of caspase3 and caspase10	Colon adenocarcinoma	Caco-2 cells	L-menthol	Faridi *et al.* Faridi et al. (2011)
					Faridi *et al.* Faridi et al. (2016)
	Via stimulation of TRPM8 channels	Prostate cancer	LNCaP cells	menthol	Berk *et al.* Beck et al. (2007)
	In liver tumor cells	Leukemia	Mouse leukemia model	(−)-menthol	Lu *et al.* Lu et al. (2007b)
Effect of menthol on cell cycle arrest	Inhibition of G0/G1 phase	Prostate cancer	DU145 cells	Menthol	Wang *et al.* Wang et al. (2012)
	Inhibition of the G2/M phase, possibly associated with polo-like kinase 1	Prostate cancer	PC-3 cells	Menthol	Kim *et al.* Kim et al. (2012)
	Inhibition of the G2/M phase and increased sub-diploid cells	Skin cancer	A431 cells	Neomenthol	Fatima *et al.* Fatima et al. (2021)
Effect of menthol on tubulin polymerization	Induction of tubulin aggregation	Colon adenocarcinoma	Caco-2 cells	L-menthol	Faridi *et al.* Faridi et al. (2011)
					Faridi *et al.* Faridi et al. (2016)
	Inhibition of tubulin aggregation	Skin cancer	A431 cells	Neomenthol	Fatima *et al.* Fatima et al. (2021)
Increase [Ca^2+^]i- mediated cell death	Activation of the calcium endocytosis pathway to increase (Ca^2+^)i	Prostate cancer	PC-3 cells	Menthol	Kim *et al.* Kim et al. (2009)
					Grolez *et al.* Grolez et al. (2019)
					Asuthkar *et al.*
					Asuthkar et al. (2015)
	Reduction of store-operated channel current	Prostate cancer	LNCaP cells	Menthol	Berk *et al.* Beck et al. (2007)
					Thebault *et al.* Thebault et al. (2005)
	Release of (Ca^2+^)i stores	Prostate cancer	LNCaP cells	Menthol	Zhang *et al.* Zhang and Barritt (2004)
	Increased TRPM8-induced (Ca^2+^) levels	Bladder cancer	T24 cells	Menthol	Li *et al.* Li et al. (2009)
	Increased (Ca^2+^)i concentration	Promyelocytic leukemia	HL60 cells	Menthol	Lu *et al.* Lu et al. (2006)
Inhibition of tumor invasion and metastasis	Activation of TRPM8 channels to inhibit cell motility	Pancreatic ductal adenocarcinoma	Panc-1 cells	Menthol	Cucu *et al.* Cucu et al. (2014)
	Down-regulation of PTK2 to reduce cell motility	Prostate cancer	DU145 cells	Menthol	Wang *et al.* Wang et al. (2012)
	Down-regulation of PTK2 to reduce cell motility	Prostate cancer	PC-3 cells	Menthol	Yang *et al.* Yang et al. (2009)
					Zhu *et al.* Zhu et al. (2011)
Inhibition of tumor angiogenesis	Via activating TRPM8 to block the trans-activation of TRPV1 by VEGF	Prostate cancer	PC-3 cells	Menthol	Zhu G *et al.* Zhu et al. (2011)
	Via inhibition of angiogenesis by activating TRPM8 to block the trans-activation of TRPV1 by VEGF	Human uveal melanoma (UM)	UM92.1 cells	Menthol	Walcher *et al.* Walcher et al. (2018)
Inhibition of tumor proliferation	By interfering with the N-acetylation of 2-aminofluorene, thus inhibiting NAT activity	Liver cancer	J5	Menthol	Lin *et al.* Lin et al. (2001)
	By promoting hematopoietic stem cell differentiation and increasing LGALS3, ITGAM, and CD19 in blood	Leukemia	Mice leukemia models	(−)-Menthol	Lu *et al.* Malumbres and Barbacid (2009)
	By inhibiting the expression of cyclooxygenase-2 and proinflammatory cytokines nuclear factor kappa B, mitogen-activated protein kinase, and extracellular signal-regulated kinase	Skin cancer	Female ICR mice	Menthol	Liu *et al.* Liu et al. (2015)
	By reducing early genotoxicity, cell proliferation, and apoptosis induced by benzo(a)pyrene	Gastric cancer	Female Swiss mice	Menthol	Santo *et al.* Santo et al. (2021)

### 3.1 Effect of menthol on cell apoptosis

Apoptosis is an orchestrated cellular process that occurs in both physiological and pathological conditions. Aberrant apoptosis is a primary mechanism underlying the pathogenesis of many diseases, including cancer, in which apoptosis occurs too infrequently resulting in undying malignant cells. Accordingly, studies have revealed that apoptosis can be an important target of anticancer agents (Wong, 2011). As an anticancer agent, menthol has been found to induce the apoptosis of cancer cells *via* several mechanisms (Fatima et al., 2021).

An experimental study assessed the anticancer activity of neomenthol in the skin cancer cell line A431 and the Ehrlich Ascites Carcinoma mouse model. The study found that neomenthol altered hyaluronidase structure and inhibited hyaluronidase activity, which prevented hyaluronic acid degradation and thus induced apoptosis (Fatima et al., 2021). In another study using Caco-2 cells, L-menthol was found to downregulate heat shock protein 90, which is a molecular chaperone that mediates the activation of disparate client proteins; It additionally activated caspase3 and caspase10. These alterations further inhibit AKT (plays a key role in multiple cellular processes such as apoptosis)-related pathways to induce the release of the pro-apoptotic factor BAD from the BAD and BCL2L1 complexes (Faridi et al., 2011; Faridi et al., 2016). In addition, another study has shown that menthol induces apoptosis in LNCaP cells by stimulating the transient receptor potential melastatin subtype 8 (TRPM8) channel (Beck et al., 2007). Lu *et al.* found that (−)-menthol induces apoptosis in liver tumor cells of BALB/c mice, in whom leukemia was induced using the mouse leukemia cells WEHI-3 (Lu et al., 2007b).

### 3.2 Effect of menthol on cell cycle arrest

The cell cycle regulates cell growth, replication of genetic material, and cell division. While the cell cycle is tightly controlled in normal cells, that in tumor cells is dysregulated owing to genetic alterations, which often results in tumors (Malumbres and Barbacid, 2009). Therefore, individual cell cycles and inhibition of cell cycle proteins have become targets for cancer therapy (Suski et al., 2021).

Menthol has been found to suppress the development of cancer cells by mediating cell cycle arrest. In the prostate cancer cell line DU145, menthol induced G0/G1 phase cell arrest (Wang et al., 2012). In another prostate cancer cell line, PC-3, menthol likely altered gene expression, inhibiting the G2/M phase; In addition, menthol may be associated with the downregulation of polo-like kinase 1, which plays an important role in the initiation, maintenance, and completion of mitosis (Kim et al., 2012). Moreover, menthol retards the growth of tumor cells in the human epidermoid carcinoma A431 cells by arresting the G2/M phase and increasing the number of sub-diploid cells (Fatima et al., 2021).

### 3.3 Effect of menthol on tubulin polymerization

Tubulin polymerization involves the assembly of the highly conserved *a*- and *ß*-tubulin heterodimers into dynamic microtubules, which perform multiple important cellular functions such as providing structural support, allowing intracellular substance transport, and generating force for cell division. Therefore, the degree of tubulin aggregation in cells is highly correlated with cell morphology and division, and alteration in this aggregation may cause cell death. Notably, tubulins are expressed in different forms by specific genes. A few tubulins are differentially expressed between normal and tumor cells, which could provide a basis for the development of anticancer drugs (Binarová and Tuszynski, 2019). Menthol has been shown to play a role in regulating tubulin in cancer cells, providing a basis for its use as an anticancer agent. Faridi *et al.* found that high concentrations of L-menthol (IC_50_ = 12 mg/mL) promoted microtubule protein polymerization, prompting cell death in Caco-2 cells (Faridi et al., 2011). In addition to L-menthol, neomenthol (100 μm) inhibits tubulin polymerization, thus arresting the proliferation of A431 skin cancer cells (Fatima et al., 2021).

### 3.4 Intracellular Ca^2+^ concentration-mediated cell death

Calcium ions (Ca^2+^) are the key secondary messengers in both excitable and non-excitable cells. There is now a large body of research showing that intracellular Ca^2+^ [(Ca^2+^)i] fluxes are associated with cancer progression and could be a potential target for cancer therapy, particularly targeting tumor growth and metastasis (Marchi et al., 2018; Marchi et al., 2020). In addition to this, research has demonstrated the involvement of Ca^2+^ in cell death (Marchi et al., 2018) as well as in the necrotic process of cancer cells (Danese et al., 2021). Menthol has been reported to induce cell necrosis primarily by upregulating (Ca^2+^)i within cancer cells *via* TRPM8, a non-selective, multimodal ion channel, activated at low temperatures (<28°C), pressure, and cooling compounds (e.g., menthol and icilin). Menthol additionally increases (Ca^2+^)i concentration in other ways, which are still unclear (Kim et al., 2009).

In the prostate cancer cell line PC-3, menthol increases (Ca^2+^)i concentration and induces cell death by activating the calcium influx pathway and other unclear channels (Kim et al., 2009; Asuthkar et al., 2015; Grolez et al., 2019). Similarly, in LNCaP cells (lymph node-derived prostate cancer cells), menthol induces cell death by reducing store-operated channel current (Thebault et al., 2005; Beck et al., 2007), thus releasing the small amount of calcium stored in the cell (Zhang and Barritt, 2004). Menthol can induce a dose-dependent increase in (Ca^2+^)i through TRPM8, resulting in the death of T24 cells (human bladder cancer cells) (Li et al., 2009). In HL60 (human promyelocytic leukemia) cells, menthol was found to increase (Ca^2+^)i concentration, thus inducing necrosis (Lu et al., 2006).

### 3.5 Inhibition of tumor invasion and metastasis

Metastatic cancer is responsible for a vast majority of cancer-related deaths and is extremely difficult to treat. Intrinsic factors both in the tumor cells and the host contribute to tumor metastasis; thus, inhibiting metastasis involves the alteration of key pathways in multiple stages of tumor progression. In addition, scientists believe that motility is a key driver of metastasis and may be used as a target for the development of anticancer treatment and prevention of metastasis (Stoletov et al., 2020). Menthol has been found to impede tumor cell motility (Wang et al., 2012). In Panc-1 cells (pancreatic ductal adenocarcinoma cells), menthol was found to significantly reduce cell motility and motility by activating TRPM8 channels (Cucu et al., 2014).

Further studies have found that in DU145 and PC-3 prostate cancer cells, menthol inhibits cell motility by inhibiting the phosphorylation of protein tyrosine kinase 2 (PTK2) through the TRMP8 pathway (Yang et al., 2009; Zhu et al., 2011; Wang et al., 2012). PTK2 is a non-receptor tyrosine kinase that is considered to play a key role in tumor cell migration, invasion, and metastasis (Lee et al., 2020).

### 3.6 Inhibition of tumor angiogenesis

In 1971, Folkman first proposed that angiogenesis is essential for the development and growth of solid tumors beyond the size of 1–2 mm (Folkman, 1971). Since then, treatments targeting angiogenesis have been considered a promising therapeutic approach for solid tumors and have been focused on by various research groups after experimental validation by several research teams (Li et al., 2018). Accordingly, researchers have focused on the anti-tumorigenic activity of menthol.

Vascular endothelial growth factor (VEGF) is one of the most potent and specific angiogenic factors that is closely associated with tumor progression (Carmeliet, 2005). Zhu *and Walcher’s et al.* found that in uveal melanoma UM92.1 cells and PC-3 cells, menthol can block the trans-activation of transient receptor potential vanilloid 1 (TRPV1;) by activating TRPM8, which further inhibits VEGF-induced angiogenesis (Zhu et al., 2011; Walcher et al., 2018).

### 3.7 Inhibition of tumor proliferation

In addition to the aforementioned six anti-cancer mechanisms, menthol can inhibit the proliferation of cancer cells by other mechanisms, such as by antagonizing cancer inducers.

In human liver cancer cells (J5), menthol was found to down regulate the N-acetylation of 2-aminofluorene by inhibiting N-acetyltransferase (NAT) activity in a dose-dependent manner (Lin et al., 2001). NAT has been reported to play a role in carcinogenesis induced by a few chemicals (Grant et al., 1991; Minchin et al., 1992). Another study demonstrated that menthol induces cytotoxicity in WEHI-3 cells in a dose-dependent manner. In BALB/c mice with leukemia (induced by WEHI-3 cells), menthol inhibited liver and spleen enlargement; increased the number of megakaryocytes in the spleen; and increased the blood levels of LGALS3, integrin alpha M, and CD19 antigen. This implies that menthol inhibits tumor development and promotes hematopoietic stem cell differentiation (Lu et al., 2007b).

In female ICR mice with skin cancer (induced by 9, 10-dimethylbenz[a]anthracene/12-O-tetradecanoylphorbol-13-acetate), menthol could inhibit tumor formation and growth in a dose-dependent manner, reducing tumor incidence and volume. Mechanistically, menthol inhibits the expression of cyclooxygenase-2 and proinflammatory cytokine nuclear factor kappa B, mitogen-activated protein kinase protein, and extracellular signal-regulated kinase, resulting in an anti-tumor effect (Liu et al., 2015). In mice, menthol demonstrated an obvious inhibitory effect on azoxymethane/dextran sulfate sodium-induced colon cancer. In another study, female Swiss mice were administered benzo(a)pyrene to induce cancer. Treatment with menthol (50 mg/kg of body weight, twice a week) at the initial stage of benzo(a)pyrene administration showed a reduced incidence of precancerous gastric lesions owing to reduced genotoxicity, reduced cell proliferation, and regulated apoptosis (Santo et al., 2021).

## 4 Critical considerations

Despite the vast evidence regarding menthol’s anticancer activity, not many human clinical trials have been conducted for this agent. Therefore, researchers are currently looking at more anticancer potentials of menthol from following perspectives.

### 4.1 Synergistic interactions of menthol with other treatments

Many natural products have synergistic effects with other listed anticancer drugs. The main advantage of combined therapy is reducing the dose and toxicity of chemotherapy while maintaining or even increasing its efficacy. Several studies have explored the advantages of combining natural compounds with classical chemotherapeutic drugs (Chen et al., 2022).

For example, menthol has been found to reduce the resistance of human hepatocellular carcinoma HepG2 cells to the anticancer drugs paclitaxel and vincristine by inhibiting the expression of cytochrome P450 family 3 subfamily A member 4 (CYP3A4), thus preventing cancer. Reportedly, CYP3A4 is implicated in the metabolism of anticancer drugs. The induction of CYP3A4 increases the metabolism of paclitaxel and vincristine, thus reducing response and eventually conferring resistance against these anticancer drugs (Yao et al., 2000; Martínez et al., 2002; Nagai et al., 2019). In addition, studies have shown that some natural compounds, such as menthol, combine with non-toxic digitalis glycosides to enhance cytotoxicity and inhibit cancer cells proliferation by downregulating the expression of ATP binding cassette subfamily B member 1 (ABCB1) (Eid et al., 2012; Eid et al., 2013).

It is worth mentioning that menthol can reduce the incidence of microadenomas by increasing the concentration of butyric acid in the feces of AD mice and increasing the abundance of bacteria. This reduces intestinal inflammation and inhibits colon cancer cell proliferation (Luo et al., 2021).

These studies provide a new perspective on the use of menthol in combination anticancer therapy. Although reports on the synergistic effects of menthol with other drugs are currently limited to those mentioned above, more potential anticancer options are waiting to be explored.

### 4.2 Clinical application

**FIGURE 4:**
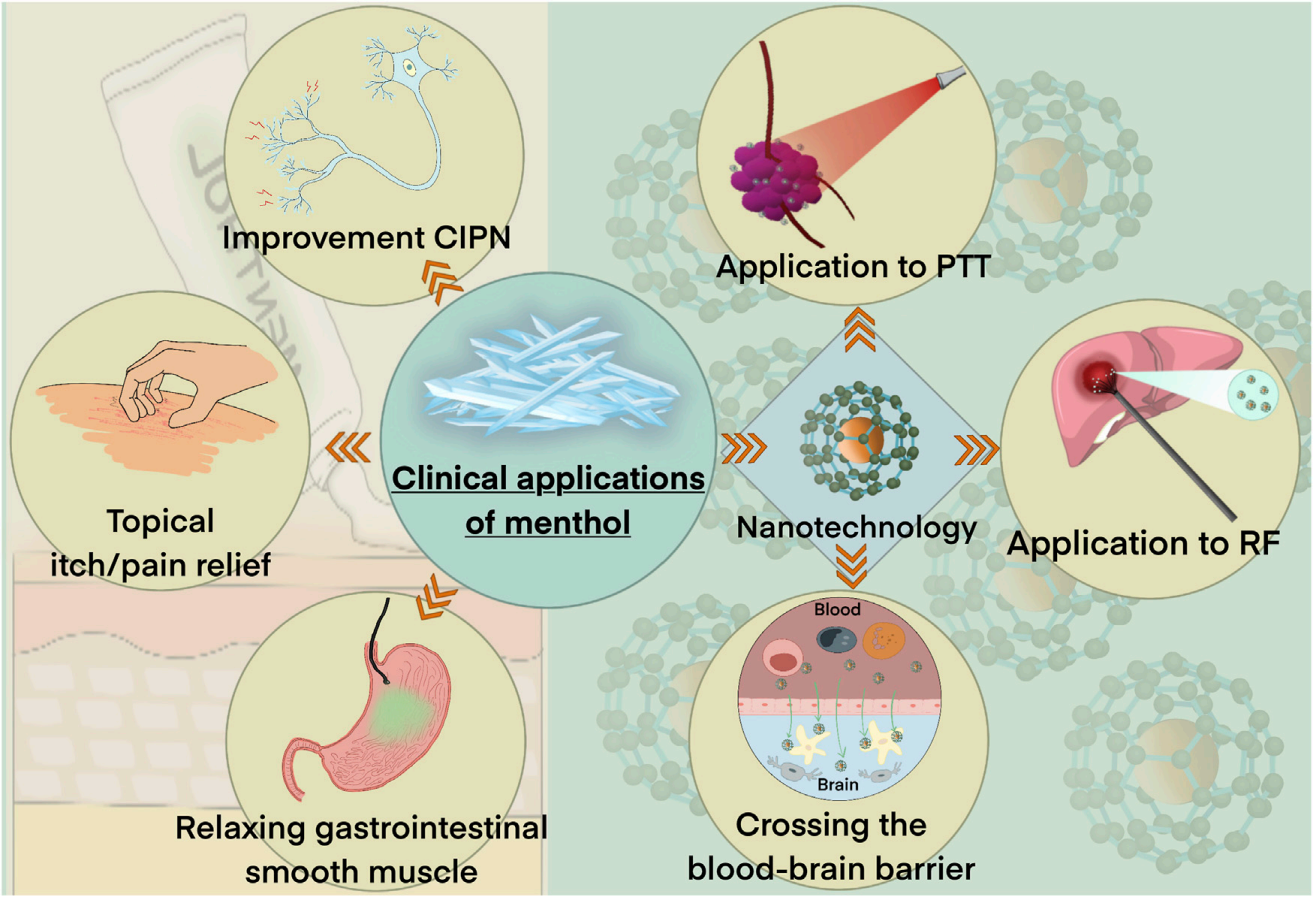
Clinical application of menthol.

As with many natural products, clinical evidence on menthol remains scarce. However, we have found that menthol continues to play an important role in some anticancer therapies (Figure 4).

Long-term treatment-related neuropathic pain due to chemotherapy-induced peripheral neuropathy (CIPN) or surgical scars is increasingly affecting the lives of patients with cancer (Cavaletti et al., 2011). A clinical study has found that the menthol receptor TRPM8 plays a role in relieving pain and may be a promising target in CIPN treatment (Singh et al., 2021). Menthol has long been used as a topical pain reliever, and modern studies have shown that it can be used as an alternative to opioid analgesics for relieving local pain (Finch and Drummond, 2015; Pergolizzi et al., 2018). Fallon *et al.* evaluated changes in pain after topical treatment with 1% menthol cream in 51 patients with CIPN (35/51) or scar pain due to cancer (10/51). They found that 82% (31/38) patients showed improvement in median pain scores [from 47 (interquartile range: 30–64) to 34 (6–59), *p* < 0.001] (Fallon et al., 2015). In another study, Cortellini *et al.* reported improvement in CIPN in a male patient following treatment with an aqueous menthol emulsion (Cortellini et al., 2017). These reports suggest that the topical use of menthol has the potential as a new analgesic in cancer-related neuropathic pain.

In addition to pain, menthol plays a special role in other cancer residual effects. For example, in an animal study of head and neck cancer, Sanz *et al.* used an Orabase^®^ platform-loading Transcutol^®^ (10%) and menthol (5%) for the buccal vehiculation of doxorubicin (DOX). Comparison with no menthol loading, this increased the residue of DOX in animal mucosa which helped better relieve oral mucositis-related pain following radiotherapy and chemotherapy (Sanz et al., 2017). Tourlaki *et al.* assessed the effect of using a detergent containing dihydroavenanthramide D 5% + 1% menthol moisturizer daily. They found that the intervention improved chronic pruritus associated with xerosis in elderly adults with Kaposi’s sarcoma (Tourlaki et al., 2020).

Previous animal studies have shown that menthol can inhibit calcium influx and potassium depolarization, resulting in reduced calcium uptake (Hawthorn et al., 1988; Hills and Aaronson, 1991; Amato et al., 2014), which relaxes gastrointestinal smooth muscles (Kim et al., 2016). In addition, a few studies have described the efficacy and safety of menthol in clinical practice (Alammar et al., 2019; Ford et al., 2008; Pittler and Ernst, 1998; Li et al., 2019; National Collaborating Centre for Nursing and Supportive Care (UK), 2008). Moreover, accumulating evidence from clinical trials has shown that directly spraying L-menthol onto the gastrointestinal mucosa significantly improves spasms; it also improves the adenoma detection rate (ADR) during an endoscopic procedure (You et al., 2020). In 2011, a study proposed that during upper gastrointestinal endoscopy, direct endoscopic spraying of L-menthol inhibits gastric peristalsis with almost no adverse reactions (Hiki et al., 2011a; Hiki et al., 2011b). Since then, more research has been conducted in this regard to validate the use of L-menthol in therapeutic endoscopies, such as endoscopic submucosal dissection of gastric tumors (Fujishiro et al., 2014; Ishiyama et al., 2021). In addition, during endoscopic surgery, L-menthol was found to effectively inhibit gastrointestinal motility, thus improve the ADR (Inoue et al., 2014; Inoue et al., 2020) and endoscopic imaging in early gastric cancer (Kikuchi et al., 2021). However, other researchers have pointed out that L-menthol could indeed alleviate adverse reactions such as gastrointestinal peristalsis and even spasm during endoscopy. Thus, the correlation between L-menthol and ADR requires further investigation (Shah et al., 2019; Aziz et al., 2020; You et al., 2020).

Recently, the timely use of menthol in some cancer treatments, such as photothermal therapy (PTT) and radiofrequency, has been found to improve treatment outcomes. Mechanistically, L-menthol-mediated heating triggers a tri-phase transition that produces continuous gas microbubbles, aiding drug release and enhanced ultrasound imaging (Zhang et al., 2016a; Zhang et al., 2016b; Feng et al., 2017; Ma et al., 2019; Shi et al., 2019; Yang et al., 2019; Zhang et al., 2019; Xu et al., 2020; Li et al., 2021). In addition, menthol is highly permeable, allowing it to deliver drugs to tumor lesions faster and more efficiently in nanotechnology applications (Liang et al., 2018; Gao et al., 2019; Liang et al., 2019; Otake et al., 2021). In one such application, gold nanoshell cerasome-encapsulated L-menthol (GNC-LM) has been shown to greatly prolong the ultrasound imaging time *in vivo* with good contrast enhancement, *via* the continuous release of gas microbubbles (Guan et al., 2019). Meanwhile, PTT based on the GNC-LM system could effectively ablate tumors, indicating the potential use of GNC-LM as a therapeutic nanoprobe for ultrasound imaging and in PTT (Guan et al., 2019). For glioma treatment, menthol-modified casein nanoparticles encapsulating anti-cancer drugs showed deeper tumor penetration and higher brain tumor distribution than unmodified nanoparticles. Thus, menthol excelled in overcoming the concern of blood-brain barrier permeability (Gao et al., 2019; Liang et al., 2019).

Although menthol is a promising anticancer drug, it needs to be further developed based on its pharmacological activity. In addition, studies on menthol, such as those related to its pharmacokinetic properties, toxicity, and bioavailability, are crucial and can provide a scientific and theoretical basis for its clinical application. Thus, studies on toxicity, combination therapy, and clinical application of menthol should be the focus of future research.

### 4.3 Limitations and controversies

Menthol has been demonstrated to have considerable anticancer activity but has been greatly restricted in clinical use because of its poorly defined toxic side effects and pharmacokinetics, in addition to other shortcomings. Despite extensive research on the role and use of menthol in the prevention and treatment of cancer, only a few studies have assessed its toxicity and pharmacokinetics. We collected the toxicity and pharmacokinetics reports of menthol from the literature, which can provide useful data for further research and development of new drugs containing menthol. Overdose of menthol administered orally (lethal dose: 50–150 mg/kg) has been known to cause convulsions, agitation, dizziness, ataxia, and coma (Opdyke, 1976). Reportedly, the median lethal dose (LD50) of L-menthol administered to mice *via* oral gavage is 3.4 g/kg, whereas the LD50 of L-menthol administered intradermally to rabbits is > 5 g/kg (Bhatia et al., 2008). Currently, no acute toxicity studies of menthol in humans have been reported. However, animal studies have shown that excessive use of L-menthol results in euphoria, coma, convulsions, and alterations in the liver and kidneys, but most of these symptoms disappear after discontinuing the agent for a while (Bhatia et al., 2008). Particularly, a study reported the case of an 86-year-old American who had been receiving a menthol-based cough suppressant for 20 years. At presentation, he was in a coma and had ataxia, recurrent oral ulcers, intermittent diarrhea, chronic dizziness, heartburn, macular skin, oliguria, and dark brown urine. However, all these symptoms disappeared after 6 months of discontinuing the cough suppressant and while receiving alternate treatment (Baibars et al., 2012). Two cases of patients presenting with coma shock possibly due to an overdose of peppermint oil were reported in India. One patient improved after hospitalization (Nath et al., 2012). However, the other patient experienced recurrent convulsions and intermittent hematuria and died after 10 days of persistent coma under hospital care (Kumar et al., 2016). Pharmacokinetic studies in humans have revealed that L-menthol (100-mg capsules) has an area under the curve of 1,214 μmol/L·min/L and a plasma half-life of 56 ± 8 min (range: 45–74 min). In addition, the peak blood concentration (C_max_) of menthol was 16.73 ± 5.53 μmol/L (range: 7.95–29.10 μmol/L), and the time to reach C_max_ was 61 ± 26 min (range: 30–120 min) (Gelal et al., 1999). In rats, the half-life of inhaled and intravenously administered L-menthol was 8.53 and 6.69 h, respectively. The peak blood concentration was noted at 1.0 h after inhalation, with an absolute lung bioavailability of 50.24% (Feng et al., 2019). These data suggest that menthol is rapidly metabolized.

Studies have reported increased (Ca^2+^)i, following menthol treatment in DBTRG cells by activating TRPM8 channels. This would further activate BK ion channels (large-conductance Ca^2+^-activated K+ channels) to promote tumor cell migration and accelerate tumor metastasis (Wondergem et al., 2008; Wondergem and Bartley, 2009). In HSC3 and HSC4 cells (oral squamous cell carcinoma cell line), menthol enhances the migration and invasion of tumor cells by inducing the activity of matrix metalloproteinase-9 (Okamoto et al., 2012). A study demonstrated that pretreatment with menthol reduces the cytotoxicity of DOX in HepG2 cells; This occurs *via* reduced concentrations of intracellular DOX following the upregulation of ABCB1 (Nagai et al., 2020). Owing to the beneficial effects of menthol and following the advent of menthol cigarettes, many people believe that smokers have been consuming more cigarettes and that menthol cigarettes increase the chances of lung cancer. However, a large body of research shows that smoking menthol cigarettes do not increase the risk of lung cancer; Instead, it may reduce the risk and post-lung cancer mortality (Richardson, 1997; Carpenter et al., 1999; Brooks et al., 2003; Blot et al., 2011; Lee, 2011; Rostron, 2012).

Although menthol is widely regarded as a safe and effective anticancer agent, it is essential to monitor its toxic side effects and be cautious when using it. Researchers should be careful about doses when developing drugs to prevent dose-related toxicity. More research is needed to further clarify the specific effects and mechanisms of menthol against different cancers, to optimize its clinical applications.

## 5 Conclusions and perspective

Plants have provided us with several pharmacologically active molecules, such as artemisinin and paclitaxel. Further research and optimization of these molecules will contribute to the betterment of human health. The effectiveness of menthol as an anticancer agent has been extensively documented. Mechanistically, menthol acts on several key molecular targets and mechanisms involved in cancer pathogenesis, indicating its potential as an anticancer agent. Menthol mediates its anticancer activity by exerting a combination of proliferative, invasive, and apoptotic effects on tumor cells as well as inhibiting tumor growth through multiple pathways. In addition to these, its low side effects makes menthol a suitable candidate for the treatment of cancer.

Despite the potential anticancer activity of menthol, it has not been widely used in human clinical trials. Accordingly, research in this direction is the need of the hour. In addition, as an underestimated anticancer agent, more studies are required to broaden the scope of traditional uses and provide better formulations, particularly those involving additional active molecules, which require more extensive and interdisciplinary efforts. Existing research has demonstrated that combining menthol with other agents can help reduce menthol dose and associated toxicity while maintaining or even increasing its efficacy. In addition, menthol has proven effective in cancer sequelae and can play a crucial adjunctive role in other therapeutic approaches like PTT and RF.

Further in-depth studies are required regarding pharmacokinetics in humans, toxicology, and mechanisms underlying combination therapy. In addition, research on new menthol-based biomaterials is required for reducing drug toxicity and developing structurally optimized analogs. All these will further enhance the efficacy and safety of menthol. Moreover, clinical studies are warranted to validate preclinical studies on the anticancer activity of menthol. This review has summarized the anticancer activity and the underlying mechanisms of menthol as well as its limitations and current problems. We believe that this review can help guide the clinical application of menthol as an anticancer drug in the future.

## References

[B1] Alammar N. Wang L. Saberi B. Nanavati J. Holtmann G. Shinohara R. T. (2019). The impact of peppermint oil on the irritable bowel syndrome: A meta-analysis of the pooled clinical data. BMC Complement. Altern. Med. 19 (1), 21. Available at: https://pubmed.ncbi.nlm.nih.gov/30654773/ . 10.1186/s12906-018-2409-0 30654773 PMC6337770

[B2] Altice C. K. Banegas M. P. Tucker-Seeley R. D. Yabroff K. R. (2016). Financial hardships experienced by cancer survivors: A systematic review. JNCI J. Natl. Cancer Inst. 109, djw205. 10.1093/jnci/djw205 27754926 PMC6075571

[B3] Alzehr A. Hulme C. Spencer A. Morgan-Trimmer S. (2022). The economic impact of cancer diagnosis to individuals and their families: A systematic review. Support Care Cancer Off. J. Multinatl. Assoc. Support Care Cancer 30, 6385–6404. 10.1007/s00520-022-06913-x PMC9213304 35235040

[B4] Amato A. Liotta R. Mulè F. (2014). Effects of menthol on circular smooth muscle of human colon: Analysis of the mechanism of action. Eur. J. Pharmacol. 740, 295–301. 10.1016/j.ejphar.2014.07.018 25046841

[B5] Api A. M. Belsito D. Botelho D. Browne D. Bruze M. Burton G. A. (2018). RIFM fragrance ingredient safety assessment, l-menthyl lactate, CAS Registry Number 59259-38-0. Food Chem. Toxicol. Int. J. Publ. Br. Ind. Biol. Res. Assoc. 115 (1), S61–S71. 10.1016/j.fct.2017.11.041 29175580

[B6] Api A. M. Belsito D. Botelho D. Browne D. Bruze M. Burton G. A. (2017). RIFM fragrance ingredient safety assessment, menthyl acetate (isomer unspecified), CAS Registry Number 16409-45-3. Food Chem. Toxicol. Int. J. Publ. Br. Ind. Biol. Res. Assoc. 110 (1), S619–S628. 10.1016/j.fct.2017.10.014 29030258

[B7] Asuthkar S. Velpula K. K. Elustondo P. A. Demirkhanyan L. Zakharian E. (2015). TRPM8 channel as a novel molecular target in androgen-regulated prostate cancer cells. Oncotarget 6, 17221–17236. 10.18632/oncotarget.3948 25980497 PMC4627303

[B8] Aziz M. Sharma S. Ghazaleh S. Fatima R. Acharya A. Ghanim M. (2020). The anti-spasmodic effect of peppermint oil during colonoscopy: A systematic review and meta-analysis. Minerva Gastroenterol. Dietol. 66, 164–171. 10.23736/S1121-421X.20.02652-5 31994371

[B9] Baibars M. Eng S. Shaheen K. Alraiyes A. H. Alraies M. C. (2012). Menthol toxicity: An unusual cause of coma. Case Rep. Med. 2012, 187039. 10.1155/2012/187039 23251165 PMC3521632

[B10] Beck B. Bidaux G. Bavencoffe A. Lemonnier L. Thebault S. Shuba Y. (2007). Prospects for prostate cancer imaging and therapy using high-affinity TRPM8 activators. Cell Calcium 41, 285–294. 10.1016/j.ceca.2006.07.002 16949669

[B11] Bhatia S. P. McGinty D. Letizia C. S. (2008). Fragrance material review on l-menthol. Food Chem. Toxicol. Int. J. Publ. Br. Ind. Biol. Res. Assoc. 46 (11), S218–S223. 10.1016/j.fct.2008.06.058 18640225

[B12] Binarová P. Tuszynski J. (2019). Tubulin: Structure, functions and roles in disease. Cells 8, 1294. 10.3390/cells8101294 31652491 PMC6829893

[B13] Blot W. J. Cohen S. S. Aldrich M. McLaughlin J. K. Hargreaves M. K. Signorello L. B. (2011). Lung cancer risk among smokers of menthol cigarettes. J. Natl. Cancer Inst. 103, 810–816. 10.1093/jnci/djr102 21436064 PMC3096798

[B14] Brooks D. R. Palmer J. R. Strom B. L. Rosenberg L. (2003). Menthol cigarettes and risk of lung cancer. Am. J. Epidemiol. 158, 609–616; discussion 617-620. 10.1093/aje/kwg182 14507595

[B15] Carmeliet P. (2005). VEGF as a key mediator of angiogenesis in cancer. Oncology 69 (3), 4–10. 10.1159/000088478 16301830

[B16] Carpenter C. L. Jarvik M. E. Morgenstern H. McCarthy W. J. London S. J. (1999). Mentholated cigarette smoking and lung-cancer risk. Ann. Epidemiol. 9, 114–120. 10.1016/s1047-2797(98)00042-8 10037555

[B17] Cavaletti G. Alberti P. Frigeni B. Piatti M. Susani E. (2011). Chemotherapy-induced neuropathy. Curr. Treat. Options Neurol. 13, 180–190. 10.1007/s11940-010-0108-3 21191824

[B18] Chen N. Qi Y. Ma X. Xiao X. Liu Q. Xia T. (2022). Rediscovery of traditional plant medicine: An underestimated anticancer drug of chelerythrine. Front. Pharmacol. 13, 906301. 10.3389/fphar.2022.906301 35721116 PMC9198297

[B19] Chrisman B. B. (1978). Menthol and dermatitis. Arch. Dermatol 114, 286. 10.1001/archderm.114.2.286c 629559

[B20] Clemente C. M. Robledo S. M. Ravetti S. (2022). Menthol carbonates as potent antiparasitic agents: Synthesis and *in vitro* studies along with computer-aided approaches. BMC Complement. Med. Ther. 22, 156. 10.1186/s12906-022-03636-8 35698116 PMC9190099

[B21] Cohen S. M. Eisenbrand G. Fukushima S. Gooderham N. J. Guengerich F. P. Hecht S. S. (2020). FEMA GRAS assessment of natural flavor complexes: Mint, buchu, dill and caraway derived flavoring ingredients. Food Chem. Toxicol. Int. J. Publ. Br. Ind. Biol. Res. Assoc. 135, 110870. 10.1016/j.fct.2019.110870 31604112

[B22] Cortellini A. Verna L. Cannita K. Napoleoni L. Parisi A. Ficorella C. (2017). Topical menthol for treatment of chemotherapy-induced peripheral neuropathy. Indian J. Palliat. Care 23, 350–352. 10.4103/ijpc.Ijpc_23_17 28827946 PMC5545968

[B23] Croteau R. B. Davis E. M. Ringer K. L. Wildung M. R. (2005). (-)-Menthol biosynthesis and molecular genetics. Naturwissenschaften 92, 562–577. 10.1007/s00114-005-0055-0 16292524

[B24] Cucu D. Chiritoiu G. Petrescu S. Babes A. Stanica L. Duda D. G. (2014). Characterization of functional transient receptor potential melastatin 8 channels in human pancreatic ductal adenocarcinoma cells. Pancreas 43, 795–800. 10.1097/MPA.0000000000000106 24658318

[B25] Danese A. Leo S. Rimessi A. Wieckowski M. R. Fiorica F. Giorgi C. (2021). Cell death as a result of calcium signaling modulation: A cancer-centric prospective. Biochim. Biophys. Acta Mol. Cell Res. 1868, 119061. 10.1016/j.bbamcr.2021.119061 33991539

[B26] Daryadel S. Atmaca U. Taslimi P. Gülçin İ. Çelik M. (2018). Novel sulfamate derivatives of menthol: Synthesis, characterization, and cholinesterases and carbonic anhydrase enzymes inhibition properties. Arch. Pharm. Weinh. 351, e1800209. 10.1002/ardp.201800209 30255953

[B27] de Mesquita L. Luz T. de Mesquita J. Coutinho D. F. Amaral F. M. M. Ribeiro M. N. (2019). Exploring the anticancer properties of essential oils from family Lamiaceae. Food Rev. Int. 35, 105–131. 10.1080/87559129.2018.1467443

[B28] Du J. Liu D. Zhang X. Zhou A. Su Y. He D. (2020). Menthol protects dopaminergic neurons against inflammation-mediated damage in lipopolysaccharide (LPS)-Evoked model of Parkinson’s disease. Int. Immunopharmacol. 85, 106679. 10.1016/j.intimp.2020.106679 32559722

[B29] Eid S. Y. El-Readi M. Z. Eldin E. Fatani S. H. Wink M. (2013). Influence of combinations of digitonin with selected phenolics, terpenoids, and alkaloids on the expression and activity of P-glycoprotein in leukaemia and colon cancer cells. Phytomedicine 21, 47–61. 10.1016/j.phymed.2013.07.019 23999162

[B30] Eid S. Y. El-Readi M. Z. Wink M. (2012). Digitonin synergistically enhances the cytotoxicity of plant secondary metabolites in cancer cells. Phytomedicine 19, 1307–1314. 10.1016/j.phymed.2012.09.002 23062361

[B31] Fallon M. T. Storey D. J. Krishan A. Weir C. J. Mitchell R. Fleetwood-Walker S. M. (2015). Cancer treatment-related neuropathic pain: Proof of concept study with menthol--a TRPM8 agonist. Support Care Cancer Off. J. Multinatl. Assoc. Support Care Cancer 23, 2769–2777. 10.1007/s00520-015-2642-8 PMC4519585 25680765

[B32] Faridi U. Dhawan S. S. Pal S. Gupta S. Shukla A. K. Darokar M. P. (2016). Repurposing L-menthol for systems medicine and cancer therapeutics? L-menthol induces apoptosis through caspase 10 and by suppressing HSP90. Omics- J. Integr. Biol. 20, 53–64. 10.1089/omi.2015.0118 PMC4739352 26760959

[B33] Faridi U. Sisodia B. S. Shukla A. K. Shukla R. K. Darokar M. P. Dwivedi U. N. (2011). Proteomics indicates modulation of tubulin polymerization by L-menthol inhibiting human epithelial colorectal adenocarcinoma cell proliferation. Proteomics 11, 2115–2119. 10.1002/pmic.201000691 21472860

[B34] Fatima K. Masood N. Ahmad Wani Z. Meena A. Luqman S. (2021). Neomenthol prevents the proliferation of skin cancer cells by restraining tubulin polymerization and hyaluronidase activity. J. Adv. Res. 34, 93–107. 10.1016/j.jare.2021.06.003 35024183 PMC8655237

[B35] Feng Q. Zhang W. Li Y. Yang X. Hao Y. Zhang H. (2017). An intelligent NIR-responsive chelate copper-based anticancer nanoplatform for synergistic tumor targeted chemo-phototherapy. Nanoscale 9, 15685–15695. 10.1039/c7nr05003h 28994432

[B36] Feng X. Liu Y. Sun X. Li A. Jiang X. Zhu X. (2019). Pharmacokinetics behaviors of l-menthol after inhalation and intravenous injection in rats and its inhibition effects on CYP450 enzymes in rat liver microsomes. Xenobiotica Fate Foreign Compd. Biol. Syst. 49, 1183–1191. 10.1080/00498254.2018.1537531 30654691

[B37] Ferlay J. Colombet M. Soerjomataram I. Parkin D. M. Piñeros M. Znaor A. (2021). Cancer statistics for the year 2020: An overview. Int. J. Cancer 149, 778–789. 10.1002/ijc.33588 33818764

[B38] Finch P. M. Drummond P. D. (2015). Topical treatment in pain medicine: From ancient remedies to modern usage. Pain Manag. 5, 359–371. 10.2217/pmt.15.23 26196538

[B39] Folkman J. (1971). Tumor angiogenesis: Therapeutic implications. N. Engl. J. Med. 285, 1182–1186. 10.1056/NEJM197111182852108 4938153

[B40] Ford A. C. Talley N. J. Spiegel B. M. R. Foxx-Orenstein A. E. Schiller L. Quigley E. M. M. (2008). Effect of fibre, antispasmodics, and peppermint oil in the treatment of irritable bowel syndrome: Systematic review and meta-analysis. BMJ 337, a2313. Available at: https://pubmed.ncbi.nlm.nih.gov/19008265/ . 10.1136/bmj.a2313 19008265 PMC2583392

[B41] Fujishiro M. Kaminishi M. Hiki N. Oda I. Fujisaki J. Uedo N. (2014). Efficacy of spraying l-menthol solution during endoscopic treatment of early gastric cancer: A phase III, multicenter, randomized, double-blind, placebo-controlled study. J. Gastroenterol. 49, 446–454. 10.1007/s00535-013-0856-4 23800946

[B42] Gao C. Liang J. Zhu Y. Ling C. Cheng Z. Li R. (2019). Menthol-modified casein nanoparticles loading 10-hydroxycamptothecin for glioma targeting therapy. Acta Pharm. Sin. B 9, 843–857. 10.1016/j.apsb.2019.01.006 31384543 PMC6663921

[B43] Gelal A. Jacob P. Yu L. Benowitz N. L. (1999). Disposition kinetics and effects of menthol. Clin. Pharmacol. Ther. 66, 128–135. 10.1053/cp.1999.v66.100455001 10460066

[B44] Gpp K. Vermaak I. Viljoen A. M. Lawrence B. M. (2013). Menthol: A simple monoterpene with remarkable biological properties. Phytochemistry 96, 15–25. 10.1016/j.phytochem.2013.08.005 24054028

[B45] Grant D. M. Blum M. Beer M. Meyer U. A. (1991). Monomorphic and polymorphic human arylamine N-acetyltransferases: A comparison of liver isozymes and expressed products of two cloned genes. Mol. Pharmacol. 39, 184–191. 1996083

[B46] Grolez G. P. Hammadi M. Barras A. Gordienko D. Slomianny C. Völkel P. (2019). Encapsulation of a TRPM8 agonist, WS12, in lipid nanocapsules potentiates PC3 prostate cancer cell migration inhibition through channel activation. Sci. Rep. 9, 7926. 10.1038/s41598-019-44452-4 31138874 PMC6538610

[B47] Guan Q. Wang C. Wu D. Wang W. Zhang C. Liu J. (2019). Cerasome-based gold-nanoshell encapsulating L-menthol for ultrasound contrast imaging and photothermal therapy of cancer. Nanotechnology 30, 015101. 10.1088/1361-6528/aae6aa 30370902

[B48] Hawthorn M. Ferrante J. Luchowski E. Rutledge A. Wei X. Y. Triggle D. J. (1988). The actions of peppermint oil and menthol on calcium channel dependent processes in intestinal, neuronal and cardiac preparations. Aliment. Pharmacol. Ther. 2, 101–118. 10.1111/j.1365-2036.1988.tb00677.x 2856502

[B49] Hiki N. Kaminishi M. Tanabe S. Fujisaki J. Yoshino J. Iguchi M. (2011). An open-label, single-arm study assessing the efficacy and safety of L: -menthol sprayed onto the gastric mucosa during upper gastrointestinal endoscopy. J. Gastroenterol. 46, 873–882. 10.1007/s00535-011-0395-9 21559772

[B50] Hiki N. Kaminishi M. Yasuda K. Uedo N. Honjo H. Matsuhashi N. (2011). Antiperistaltic effect and safety of L-menthol sprayed on the gastric mucosa for upper GI endoscopy: A phase III, multicenter, randomized, double-blind, placebo-controlled study. Gastrointest. Endosc. 73, 932–941. 10.1016/j.gie.2010.12.013 21353674

[B51] Hilfiger L. Triaux Z. Marcic C. Héberlé E. Emhemmed F. Darbon P. (2021). Anti-hyperalgesic properties of menthol and pulegone. Front. Pharmacol. 12, 753873. 10.3389/fphar.2021.753873 34916937 PMC8670501

[B52] Hills J. M. Aaronson P. I. (1991). The mechanism of action of peppermint oil on gastrointestinal smooth muscle. An analysis using patch clamp electrophysiology and isolated tissue pharmacology in rabbit and Guinea pig. Gastroenterology 101, 55–65. 10.1016/0016-5085(91)90459-x 1646142

[B53] Inoue K. Dohi O. Gen Y. Jo M. Mazaki T. Tokita K. (2014). L-Menthol improves adenoma detection rate during colonoscopy: A randomized trial. Endoscopy 46, 196–202. 10.1055/s-0034-1365035 24573731

[B54] Inoue K. Okuda T. Oka K. Sugino S. Endo Y. Ota T. (2020). Effects of L-menthol and carbon dioxide on the adenoma detection rate during colonoscopy: L-menthol and carbon dioxide on colonoscopy. Digestion 101, 323–331. 10.1159/000498941 30844795

[B55] Ishiyama A. Namikawa K. Tokai Y. Yoshimizu S. Horiuchi Y. Yoshio T. (2021). Effect of spraying l-menthol on peristalsis resumption during endoscopic submucosal dissection of gastric tumors. JGH Open Open Access J. Gastroenterol. Hepatol. 5, 653–657. 10.1002/jgh3.12549 PMC8171147 34124381

[B56] Kanezaki M. Terada K. Ebihara S. (2021). l-Menthol - a new treatment for breathlessness? Curr. Opin. Support Palliat. Care 15, 233–238. 10.1097/SPC.0000000000000569 34762073

[B57] Kikuchi H. Hikichi T. Watanabe K. Nakamura J. Hashimoto M. Kato T. (2021). Effectiveness of L-menthol spray application on lesions for the endoscopic clarification of early gastric cancer: Evaluation by the color difference. Digestion 102, 274–282. 10.1159/000504667 31822003

[B58] Kim H. J. Wie J. So I. Jung M. H. Ha K-T. Kim B. J. (2016). Menthol modulates pacemaker potentials through TRPA1 channels in cultured interstitial cells of cajal from murine small intestine. Cell Physiol. Biochem Int. J. Exp. Cell Physiol. Biochem Pharmacol. 38, 1869–1882. 10.1159/000445549 27160463

[B59] Kim S-H. Lee S. Piccolo S. R. Allen-Brady K. Park E-J. Chun J. N. (2012). Menthol induces cell-cycle arrest in PC-3 cells by down-regulating G2/M genes, including polo-like kinase 1. Biochem Biophys. Res. Commun. 422, 436–441. 10.1016/j.bbrc.2012.05.010 22580005

[B60] Kim S-H. Nam J-H. Park E-J. Kim B-J. Kim S-J. So I. (2009). Menthol regulates TRPM8-independent processes in PC-3 prostate cancer cells. Biochim. Biophys. Acta 1792, 33–38. 10.1016/j.bbadis.2008.09.012 18955132

[B61] Kumar A. Baitha U. Aggarwal P. Jamshed N. (2016). A fatal case of menthol poisoning. Int. J. Appl. Basic Med. Res. 6, 137–139. 10.4103/2229-516X.179015 27127746 PMC4830155

[B62] Lee P. N. (2011). Systematic review of the epidemiological evidence comparing lung cancer risk in smokers of mentholated and unmentholated cigarettes. BMC 11, 18. 10.1186/1471-2466-11-18 PMC3103484 21501470

[B63] Lee S. Jeon Y-M. Cha S. J. Kim S. Kwon Y. Jo M. (2020). PTK2/FAK regulates UPS impairment via SQSTM1/p62 phosphorylation in TARDBP/TDP-43 proteinopathies. Autophagy 16, 1396–1412. 10.1080/15548627.2019.1686729 31690171 PMC7469499

[B64] Li J. Lv L. Zhang J. Xu L. Zeng E. Zhang Z. (2019). A combination of peppermint oil and caraway oil for the treatment of functional dyspepsia: A systematic review and meta-analysis. Evid-Based Complement. Altern. Med. ECAM 2019, 7654947. 10.1155/2019/7654947 PMC6885176 31827561

[B65] Li M. Gao X. Lin C. Shen A. Luo J. Ji Q. (2021). An intelligent responsive macrophage cell membrane-camouflaged mesoporous silicon nanorod drug delivery system for precise targeted therapy of tumors. J. Nanobiotechnology 19, 336. 10.1186/s12951-021-01082-1 34689763 PMC8543955

[B66] Li Q. Wang X. Yang Z. Wang B. Li S. (2009). Menthol induces cell death via the TRPM8 channel in the human bladder cancer cell line T24. Oncology 77, 335–341. 10.1159/000264627 19955836

[B67] Li T. Kang G. Wang T. Huang H. (2018). Tumor angiogenesis and anti-angiogenic gene therapy for cancer. Oncol. Lett. 16, 687–702. 10.3892/ol.2018.8733 29963134 PMC6019900

[B68] Liang J. Gao C. Zhu Y. Ling C. Wang Q. Huang Y. (2018). Natural brain penetration enhancer-modified albumin nanoparticles for glioma targeting delivery. ACS Appl. Mater Interfaces 10, 30201–30213. 10.1021/acsami.8b11782 30113810

[B69] Liang J. M. Zhu Y. Gao C. F. Ling C. L. Qin J. Wang Q. (2019). Menthol-modified BSA nanoparticles for glioma targeting therapy using an energy restriction strategy. Npg Asia Mater 11, 38. 10.1038/s41427-019-0138-6

[B70] Lin J. P. Li Y. C. Lin W. C. Hsieh C. L. Chung J. G. (2001). Effects of (-)-menthol on arylamine N-acetyltransferase activity in human liver tumor cells. Am. J. Chin. Med. 29, 321–329. 10.1142/S0192415X01000344 11527074

[B71] Liu Z. Shen C. Tao Y. Wang S. Wei Z. Cao Y. (2015). Chemopreventive efficacy of menthol on carcinogen-induced cutaneous carcinoma through inhibition of inflammation and oxidative stress in mice. Food Chem. Toxicol. Int. J. Publ. Br. Ind. Biol. Res. Assoc. 82, 12–18. 10.1016/j.fct.2015.04.025 25956868

[B72] Lu H-F. Hsueh S-C. Yu F-S. Yang J-S. Tang N-Y. Chen S-C. (2006). The role of Ca2+ in (-)-menthol-induced human promyelocytic leukemia HL-60 cell death. Vivo Athens Greece 20, 69–75. 16433031

[B73] Lu H-F. Liu J-Y. Hsueh S-C. Yang Y-Y. Yang J-S. Tan T-W. (2007). (–)-Menthol inhibits WEHI-3 leukemia cells *in vitro* and *in vivo* . Vivo 21 (2), 285–289. 17436578

[B74] Lu H-F. Liu J-Y. Hsueh S-C. Yang Y-Y. Yang J-S. Tan T-W. (2007). (–)-Menthol inhibits WEHI-3 leukemia cells *in vitro* and *in vivo* . Vivo 21, 285–289. 17436578

[B75] Luo L. Yan J. Chen B. Luo Y. Liu L. Sun Z. (2021). The effect of menthol supplement diet on colitis-induced colon tumorigenesis and intestinal microbiota. Am. J. Transl. Res. 13, 38–56. 33527007 PMC7847519

[B76] Ma Y. Zhou J. Miao Z. Qian H. Zha Z. (2019). Dl-menthol loaded polypyrrole nanoparticles as a controlled diclofenac delivery platform for sensitizing cancer cells to photothermal therapy. ACS Appl. Bio Mater 2, 848–855. 10.1021/acsabm.8b00687 35016288

[B77] Malik D. Mahendiratta S. Kaur H. Medhi B. (2021). Futuristic approach to cancer treatment. Gene 805, 145906. 10.1016/j.gene.2021.145906 34411650

[B78] Malumbres M. Barbacid M. (2009). Cell cycle, CDKs and cancer: A changing paradigm. Nat. Rev. Cancer 9, 153–166. 10.1038/nrc2602 19238148

[B79] Marchi S. Giorgi C. Galluzzi L. Pinton P. (2020). Ca2+ fluxes and cancer. Mol. Cell 78, 1055–1069. 10.1016/j.molcel.2020.04.017 32559424

[B80] Marchi S. Patergnani S. Missiroli S. Morciano G. Rimessi A. Wieckowski M. R. (2018). Mitochondrial and endoplasmic reticulum calcium homeostasis and cell death. Cell Calcium 69, 62–72. 10.1016/j.ceca.2017.05.003 28515000

[B81] Martínez C. García-Martín E. Pizarro R. M. García-Gamito F. J. Agúndez J. a. G. (2002). Expression of paclitaxel-inactivating CYP3A activity in human colorectal cancer: Implications for drug therapy. Br. J. Cancer 87, 681–686. 10.1038/sj.bjc.6600494 12237780 PMC2364247

[B82] Minchin R. F. Reeves P. T. Teitel C. H. McManus M. E. Mojarrabi B. Ilett K. F. (1992). N- and O-acetylation of aromatic and heterocyclic amine carcinogens by human monomorphic and polymorphic acetyltransferases expressed in COS-1 cells. Biochem Biophys. Res. Commun. 185, 839–844. 10.1016/0006-291X(92)91703-S 1627140

[B83] Nagai K. Fukuno S. Miura T. Uchino Y. Sehara N. Konishi H. (2020). Reduced cytotoxicity in doxorubicin-exposed HepG2 cells pretreated with menthol due to upregulation of P-glycoprotein. Pharmazie 75 (10), 510–511. 10.1691/ph.2020.0448 33305727

[B84] Nagai K. Fukuno S. Omachi A. Omotani S. Hatsuda Y. Myotoku M. (2019). Enhanced anti-cancer activity by menthol in HepG2 cells exposed to paclitaxel and vincristine: Possible involvement of CYP3A4 downregulation. Drug Metab. Pers. Ther. 34, 34. 10.1515/dmpt-2018-0029 30840584

[B85] Nath S. S. Pandey C. Roy D. (2012). A near fatal case of high dose peppermint oil ingestion- Lessons learnt. Indian J. Anaesth. 56, 582–584. 10.4103/0019-5049.104585 23325948 PMC3546250

[B86] National Collaborating Centre for Nursing and Supportive Care (UK) (2008). Irritable bowel syndrome in adults: Diagnosis and management of irritable bowel syndrome in primary care. London: Royal College of Nursing. Available at: http://www.ncbi.nlm.nih.gov/books/NBK51953/ (Accessed October 22, 2022). 21656972

[B87] Newman D. J. Cragg G. M. (2020). Natural products as sources of new drugs over the nearly four decades from 01/1981 to 09/2019. J. Nat. Prod. 83, 770–803. 10.1021/acs.jnatprod.9b01285 32162523

[B88] Okamoto Y. Ohkubo T. Ikebe T. Yamazaki J. (2012). Blockade of TRPM8 activity reduces the invasion potential of oral squamous carcinoma cell lines. Int. J. Oncol. 40, 1431–1440. 10.3892/ijo.2012.1340 22267123

[B89] Opdyke D. L. (1976). Monographs on fragrance raw materials. Food Cosmet. Toxicol. 14, 307–338. 10.1016/s0015-6264(76)80295-7 976888

[B90] Otake H. Yamaguchi M. Ogata F. Deguchi S. Yamamoto N. Sasaki H. (2021). Energy-dependent endocytosis is responsible for skin penetration of formulations based on a combination of indomethacin nanoparticles and l-menthol in rat and gottingen minipig. Int. J. Mol. Sci. 22, 5137. 10.3390/ijms22105137 34066280 PMC8152063

[B91] Patel T. Yosipovitch G. (2010). Therapy of pruritus. Expert Opin. Pharmacother. 11, 1673–1682. 10.1517/14656566.2010.484420 20426711 PMC2885583

[B92] Pergolizzi J. V. Taylor R. LeQuang J-A. Raffa R. B. NEMA Research Group (2018). The role and mechanism of action of menthol in topical analgesic products. J. Clin. Pharm. Ther. 43, 313–319. 10.1111/jcpt.12679 29524352

[B93] Pittler M. H. Ernst E. (1998). Peppermint oil for irritable bowel syndrome: A critical review and metaanalysis. Am. J. Gastroenterol. 93, 1131–1135. 10.1111/j.1572-0241.1998.00343.x 9672344

[B94] Richardson T. L. (1997). African-American smokers and cancers of the lung and of the upper respiratory and digestive tracts. West J. Med. 166 (3), 189–194. 9143194 PMC1304117

[B95] Rostron B. (2012). Lung cancer mortality risk for U.S. menthol cigarette smokers. Nicotine Tob. Res. Off. J. Soc. Res. Nicotine Tob. 14, 1140–1144. 10.1093/ntr/nts014 22387991

[B96] Santo S. G. E. Romualdo G. R. Santos L. A. Grassi T. F. Barbisan L. F. (2021). Modifying effects of menthol against benzo(a)pyrene‐induced forestomach carcinogenesis in female Swiss mice. Environ. Toxicol. 36, 2245–2255. 10.1002/tox.23338 34331502

[B97] Sanz R. Calpena A. C. Mallandrich M. Gimeno A. Halbaut L. Clares B. (2017). Development of a buccal doxepin platform for pain in oral mucositis derived from head and neck cancer treatment. Eur. J. Pharm. Biopharm. 117, 203–211. 10.1016/j.ejpb.2017.04.019 28438551

[B98] Shah I. Baffy N. J. Horsley-Silva J. L. Langlais B. T. Ruff K. C. (2019). Peppermint oil to improve visualization in screening colonoscopy: A randomized controlled clinical trial. Gastroenterol. Res. 12, 141–147. 10.14740/gr1180 PMC6575129 31236155

[B99] Shi C. E. You C. Q. Pan L. (2019). Facile formulation of near-infrared light-triggered hollow mesoporous silica nanoparticles based on mitochondria targeting for on-demand chemo/photothermal/photodynamic therapy. Nanotechnology 30, 325102. 10.1088/1361-6528/ab1367 30913541

[B100] Silva H. (2020). Current Knowledge on the vascular effects of menthol. Front. Physiol. 11, 298. 10.3389/fphys.2020.00298 32317987 PMC7154148

[B101] Singh H. Kumar R. Mazumder A. Salahuddin N. Yadav R. K. Chauhan B. (2022). Camphor and menthol as anticancer agents: Synthesis, structure-activity relationship and interaction with cancer cell lines. Anticancer Agents Med. Chem. 23, 614–623. 10.2174/1871520622666220810153735 35950244

[B102] Singh R. Adhya P. Sharma S. S. (2021). Redox-sensitive TRP channels: A promising pharmacological target in chemotherapy-induced peripheral neuropathy. Expert Opin. Ther. Targets 25, 529–545. 10.1080/14728222.2021.1956464 34289785

[B103] Stoletov K. Beatty P. H. Lewis J. D. (2020). Novel therapeutic targets for cancer metastasis. Expert Rev. Anticancer Ther. 20, 97–109. 10.1080/14737140.2020.1718496 31997674

[B104] Suski J. M. Braun M. Strmiska V. Sicinski P. (2021). Targeting cell-cycle machinery in cancer. Cancer Cell 39, 759–778. 10.1016/j.ccell.2021.03.010 33891890 PMC8206013

[B105] Thebault S. Lemonnier L. Bidaux G. Flourakis M. Bavencoffe A. Gordienko D. (2005). Novel role of cold/menthol-sensitive transient receptor potential melastatine family member 8 (TRPM8) in the activation of store-operated channels in LNCaP human prostate cancer epithelial cells. J. Biol. Chem. 280, 39423–39435. 10.1074/jbc.M503544200 16174775

[B106] Tourlaki A. Genovese G. Consonni D. Brambilla L. (2020). Efficacy of a detergent combined with a moisturizer for the treatment of pruritus associated with xerosis in an elderly population affected by Kaposi’s sarcoma. G. Ital. Dermatol Venereol. 155, 487–491. 10.23736/s0392-0488.18.05765-6 29417794

[B107] Walcher L. Budde C. Böhm A. Reinach P. S. Dhandapani P. Ljubojevic N. (2018). TRPM8 activation via 3-iodothyronamine blunts VEGF-induced transactivation of TRPV1 in human uveal melanoma cells. Front. Pharmacol. 9, 1234. 10.3389/fphar.2018.01234 30483120 PMC6243059

[B108] Wang Y. Wang X. Yang Z. Zhu G. Chen D. Meng Z. (2012). Menthol inhibits the proliferation and motility of prostate cancer DU145 cells. Pathol. Oncol. Res. 18, 903–910. 10.1007/s12253-012-9520-1 22437241

[B109] Wondergem R. Bartley J. W. (2009). Menthol increases human glioblastoma intracellular Ca2+, BK channel activity and cell migration. J. Biomed. Sci. 16, 90. 10.1186/1423-0127-16-90 19778436 PMC2758849

[B110] Wondergem R. Ecay T. W. Mahieu F. Owsianik G. Nilius B. (2008). HGF/SF and menthol increase human glioblastoma cell calcium and migration. Biochem Biophys. Res. Commun. 372, 210–215. 10.1016/j.bbrc.2008.05.032 18485891

[B111] Wong R. S. Y. (2011). Apoptosis in cancer: From pathogenesis to treatment. J. Exp. Clin. Cancer Res. CR 30, 87. 10.1186/1756-9966-30-87 21943236 PMC3197541

[B112] Xu W. Peng Y. D. Zhang H. Liu L. Li J. (2020). Fabrication of novel gold nanoparticles decorated cerasome for ultrasound contrast imaging and photothermal evaluation for cancer treatment. J. Clust. Sci. 31, 805–810. 10.1007/s10876-019-01687-5

[B113] Yang X. Wang C. N. Ding M. L. Zhang C. Y. Wang W. Cao Z. (2019). Indocyanine green (ICG) - menthol loaded cerasomal nanoparticles for ultrasound imaging and photothermal therapy against tumor. Mater Lett. 255, 126524. 10.1016/j.matlet.2019.126524

[B114] Yang Z-H. Wang X-H. Wang H-P. Hu L-Q. (2009). Effects of TRPM8 on the proliferation and motility of prostate cancer PC-3 cells. Asian J. Androl. 11, 157–165. 10.1038/aja.2009.1 19234481 PMC3735032

[B115] Yao D. Ding S. Burchell B. Wolf C. R. Friedberg T. (2000). Detoxication of vinca alkaloids by human P450 CYP3A4-mediated metabolism: Implications for the development of drug resistance. J. Pharmacol. Exp. Ther. 294, 387–395. 10871337

[B116] You Q. Li L. Chen H. Chen L. Chen X. Liu Y. (2020). L-menthol for gastrointestinal endoscopy: A systematic review and meta-analysis. Clin. Transl. Gastroenterol. 11, e00252. 10.14309/ctg.0000000000000252 33031198 PMC7544180

[B117] Zhang C. Liu J. Guo H. Wang W. Xu M. Tan Y. (2019). Theranostic nanomedicine carrying L-menthol and near-infrared dye for multimodal imaging-guided photothermal therapy of cancer. Adv. Heal Mater 8, e1900409. 10.1002/adhm.201900409 31148393

[B118] Zhang K. Li P. Chen H. Bo X. Li X. Xu H. (2016). Continuous cavitation designed for enhancing radiofrequency ablation via a special radiofrequency solidoid vaporization process. ACS Nano 10, 2549–2558. 10.1021/acsnano.5b07486 26800221

[B119] Zhang K. Li P. He Y. P. Bo X. W. Li X. L. Li D. D. (2016). Synergistic retention strategy of RGD active targeting and radiofrequency-enhanced permeability for intensified RF & chemotherapy synergistic tumor treatment. Biomaterials 99, 34–46. 10.1016/j.biomaterials.2016.05.014 27209261

[B120] Zhang L. Barritt G. J. (2004). Evidence that TRPM8 is an androgen-dependent Ca ^2+^ channel required for the survival of prostate cancer cells. Cancer Res. 64, 8365–8373. 10.1158/0008-5472.CAN-04-2146 15548706

[B121] Zhang Z. Wu X. Zhang L. Mao A. Ma X. He D. (2020). Menthol relieves acid reflux inflammation by regulating TRPV1 in esophageal epithelial cells. Biochem Biophys. Res. Commun. 525, 113–120. 10.1016/j.bbrc.2020.02.050 32081421

[B122] Zhu G. Wang X. Yang Z. Cao H. Meng Z. Wang Y. (2011). Effects of TRPM8 on the proliferation and angiogenesis of prostate cancer PC-3 cells *in vivo* . Oncol. Lett. 2, 1213–1217. 10.3892/ol.2011.410 22848290 PMC3406498

[B123] Zielińska-Błajet M. Pietrusiak P. Feder-Kubis J. (2021). Selected monocyclic monoterpenes and their derivatives as effective anticancer therapeutic agents. Int. J. Mol. Sci. 22, 4763. 10.3390/ijms22094763 33946245 PMC8124601

